# Histone acetyltransferase and Polo-like kinase 3 inhibitors prevent rat galactose-induced cataract

**DOI:** 10.1038/s41598-019-56414-x

**Published:** 2019-12-27

**Authors:** Fumito Kanada, Yoshihiro Takamura, Seiji Miyake, Kazuma Kamata, Mayumi Inami, Masaru Inatani, Masaya Oki

**Affiliations:** 1 Department of Applied Chemistry and Biotechnology, Graduate School of Engineering, University of Fukui, Fukui, 910-8507 Japan; 2 Department of Ophthalmology, Faculty of Medical Sciences, University of Fukui, Fukui, 910-1193 Japan; 3 Life Science Innovation Center, University of Fukui, Fukui, 910-8507 Japan

**Keywords:** Epigenetics, Transcription

## Abstract

Diabetic cataracts can occur at an early age, causing visual impairment or blindness. The detailed molecular mechanisms of diabetic cataract formation remain incompletely understood, and there is no well-documented prophylactic agent. Galactose-fed rats and *ex vivo* treatment of lenses with galactose are used as models of diabetic cataract. To assess the role of histone acetyltransferases, we conducted cataract prevention screening with known histone acetyltransferase (HAT) inhibitors. *Ex vivo* treatment with a HAT inhibitor strongly inhibited the formation of lens turbidity in high-galactose conditions, while addition of a histone deacetylase (HDAC) inhibitor aggravated turbidity. We conducted a microarray to identify genes differentially regulated by HATs and HDACs, leading to discovery of a novel cataract causative factor, Plk3. *Plk3* mRNA levels correlated with the degree of turbidity, and Plk3 inhibition alleviated galactose-induced cataract formation. These findings indicate that epigenetically controlled Plk3 influences cataract formation. Our results demonstrate a novel approach for prevention of diabetic cataract using HAT and Plk3 inhibitors.

## Introduction

Most cataracts are age-related, and nearly 100% of people develop cataracts by 80 years of age^1^. Diabetes is also a major cause of cataracts, and patients with diabetes develop cataracts earlier and approximately five times more frequently^2^. As the global incidence of diabetes continues to increase, the incidence of diabetic cataracts will increase and become a major cause of early-onset visual impairment. The only available cataract treatment is surgical removal of the crystalline lens and replacement with a intraocular lens, but this approach is associated with significant adverse effects, and is not available in remote locations. Therefore, extending the time to required lens replacement is clinically desirable, but there are currently no known pharmacological interventions for diabetic cataract^3^.

Increased intracellular osmotic pressure due to accumulation of polyol and lens epithelial cell (LEC) apoptosis are major contributing pathologies of diabetic cataract. High intracellular osmotic pressure promotes lens turbidity by increasing cellular swelling and subsequent lens cortex degradation^4,5^, which can be prevented by inhibiting polyol accumulation using aldose reductase inhibitors^6^. On the other hand, addition of hydrogen peroxide to induce LEC apoptosis also causes cortical cataracts and ultimate collapse of the lens cortex^7^. Prior studies have also demonstrated that polyol accumulation increases LEC apoptosis, suggesting that these factors are interrelated^8^. Although the cellular events contributing to diabetic cataract formation have been reported^4–6,8^, the detailed molecular mechanisms for these events remain incompletely understood. Elucidating the molecular events of cataract formation is important to identify therapeutic interventions to prevent or delay diabetic cataract formation.

DNA sequence-independent gene expression regulation mechanisms, collectively known as epigenetics, regulate diverse physiological and pathological phenomena, and epigenetic regulators may therefore be viable therapeutic targets for many disease processes^9^. Epigenetic modifications are regulated in part by DNA methylation and posttranslational histone modification, both of which can either repress or promote gene transcription^10^. In age-related cataract, hypermethylation-mediated transcriptional repression of the antioxidant enzyme *Gstm3* promoter region has been confirmed and may contribute to cataract formation^11^. In diabetic cataract, the antioxidant inhibitor *Keap1* is transcriptionally activated by hypomethylation of the promoter region^12^. Further, methylation rates of *Cryaa*, *Ercc6*, and *Ogg1* are also changed in cataracts^13–15^. Additionally, posttranslational histone modification, especially by acetylation, may also be associated with cataract formation. Generally, histone acetyltransferase relaxes the chromatin structure through histone acetylation, increasing expression of neighboring genes. HATs are classified according to functional roles and structural features, including Pcaf and Gcn5 in the GNAT family, Tip60 and Moz in the MIST family, and p300 and Cbp in the p300/Cbp family^16^. Several prior studies investigated the relationship between histone acetylation and other types of cataract formation. UV exposure is a known risk for cataracts, and UV-B irradiation of LECs induces histone deacetylation^14^. Rong *et al*. reported that when the rabbit lens was treated with the HAT inhibitor anacardic acid, white turbidity formed on the lens surface^17^. Moreover, this turbidity did not form when anacardic acid and the histone deacetylase (HDAC) inhibitor Trichostatin A (TSA) were applied in co-treatment. Tgf-β induced cataracts in rats also causes lens surface turbidity, which is similarly inhibited by TSA^18,19^. These reports suggest that epigenetic modifications are involved in cataract formation, and that preventing these modifications suppresses cataract formation. However, the roles of epigenetic modifications in diabetic cataract are unknown.

In the present study, to evaluate the preventive effects of HAT and HDAC inhibitors in rat diabetic-like cataracts, *ex vivo* lens turbidity was induced by incubation in galactose-containing media. We then evaluated whether HDAC/HAT inhibitors affected lens turbidity in this model. Subsequently, we used microarray analysis of rat lenses ± HAT/HDAC inhibitors to identify the target gene *Plk3*, which contributes to galactose-induced cataract formation. These results identified a novel mechanism of HAT regulation of glycated cataract through Plk3.

## Results

### HAT inhibitors prevent diabetic-like cataracts

**Figure 1:**
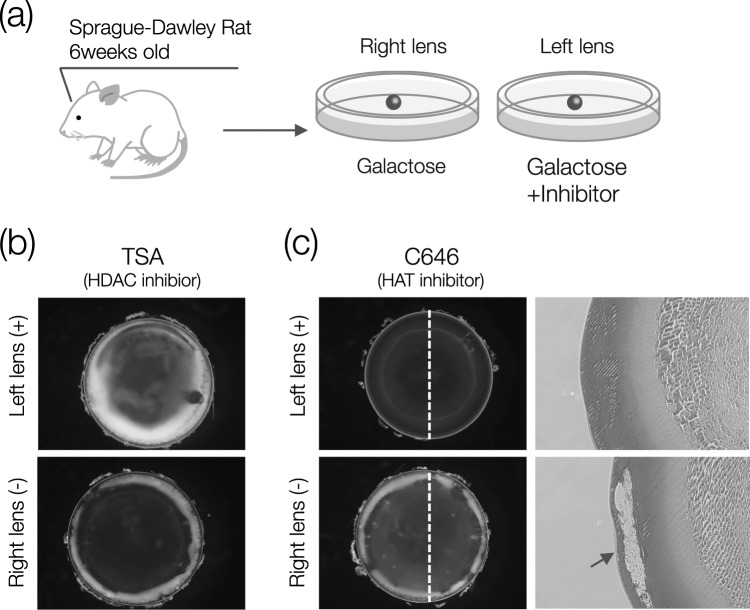
HAT inhibition alleviates galactose-induced cataract formation. (**a**) Diagram of *ex vivo* experiment. The inhibitor was added to the left lens (with the right lens serving as a Ctrl) and incubated for 4 days in medium containing 30 mM galactose. (**b**) Representative photomicrograph of lenses on Day 4 after addition of 0.8 μM TSA or vehicle. (**c**) Representative histological photographs of lenses at 40 μM C646 (Day 4). Lenses tissue sections cut in the direction of the broken line were stained with hematoxylin-eosin. Only the right lens (Ctrl group) shows large vacuoles, indicated by arrows.

We initially investigated the effect of HDAC inhibitors on lens turbidity to assess the role of histone acetylation in diabetic-like cataract formation. The rat crystalline lens was cultured in galactose-containing medium as a diabetic cataract model (Fig. 1a). The galactose cataract model is suitable for inhibitor screening because cataract formation occurs rapidly and reliably. When lenses were cultured in medium containing 30 mM galactose for 4 days, cortical cataract formation occurred, and was further increased by addition of the HDAC inhibitor TSA (Fig. 1b). This suggested a relationship between histone acetylation and lens turbidity, prompting us to next determine the effect of HAT inhibitors on cataract formation. We tested 26 different inhibitors targeting various HATs (Table 1)^20–31^, and found that 16 of the 26 tested inhibitors decreased cataract formation (Supplementary Fig. S1). The HAT inhibitors embelin and ECGC are slightly cloudy, but we determined that these inhibitors prevented cataract formation because lens turbidity was significantly reduced compared with the vehicle control. Interestingly, specific inhibitors such as TH1834 (Tip60), Embelin (Pcaf), CPTH2 (Gcn5), and C646 (p300) had similar efficacies. However, some HAT inhibitors were ineffective (Supplementary Fig. S2). To observe the inside of the lens, a section was prepared and stained with hematoxylin and eosin. In the vehicle control (Ctrl) lens exposed to galactose, numerous vacuoles were found in the equator, and the cortex was largely collapsed (Fig. 1c). On the other hand, the p300 inhibitor C646 almost completely prevented tissue collapse and maintained normal lens tissue (Fig. 1c).

**Table 1:** HAT inhibitor list.

Name	Target	Place of purchase	Concentraion	Prevent effect	Source
CTK7A	p300/PCAF	EMD Millipore (USA)	100 μM	○	(20)
Garcinol	p300/PCAF	Abcam (UK)	2 μM	○	(20,21)
Anacardic Acid	p300/PCAF	Abcam (UK)	20 μM	○	(20,21)
MG149	TIP60/MOZ	Selleck Chemicals (USA)	50 μM	○	(22)
C646	p300	Sigma Aldrich (USA)	40 μM	○	(20,21)
CPTH2	GCN5	Cayman Chemical (USA)	80 μM	○	(23)
gallic acid	HAT	Sigma Aldrich (USA)	100 μM	○	(24)
(−)-Epigallocatechin gallate (ECGC)	HAT	Wako (Japan)	50 μM	○	(20,21)
EML425	p300/CBP	Tocris (UK)	200 μM	○	(20)
ISOX DUAL	CBP/BRD4	Sigma Aldrich (USA)	20 μM	○	(25)
Plumbagin	p300	Sigma Aldrich (USA)	2 μM	○	(20,21)
TH1834	TIP60	Axon Medchem (Netherlands)	50 μM	○	(20)
windorphen	HAT	Sigma Aldrich (USA)	40 μM	○	(26)
Remodelin	NAT10	Cayman Chemical (USA)	40 μM	○	(27)
Embelin	PCAF	Abcam (UK)	40 μM	○	(20)
CBP30	p300	Cayman Chemical (USA)	5 μM	○	(20)
Curcumin	p300/CBP	Abcam (UK)	40 μM	×	(20,21)
2,6-bis((e)-3-bromo-4-hydroxybenzylidene) cyclohexan (HAT inhibitor)	p300	Abcam (UK)	40 μM	×	(28)
Epigenetic Multiple Ligand	p300/CBP	Santa Cruz (USA)	50 μM	×	(29)
NU9056	TIP60	Santa Cruz (USA)	100 μM	×	(30)
Butyrolactone 3 (MB-3)	GCN5	Abcam (UK)	200 μM	×	(20,21)
L002	p300/CBP	Cayman Chemical (USA)	10 μM	×	(20)
SPV106	p300/CBP	Sigma Aldrich (USA)	50 μM	×	(31)
Chetomin	HIF1a/p300 complex	Cayman Chemical(USA)	50 nM	×	(21)
Ischemin	p300/CBP	Tocris (UK)	100 μM	×	(20,21)
KG501(Naphthol AS-E phosphate)	p300/CBP	Sigma-Aldrich (USA)	50 μM	×	(21)

The list summarizes the prophylactic effect of 26 HAT inhibitors. Concentration indicates the concentration of the drug used.

**Figure 2:**
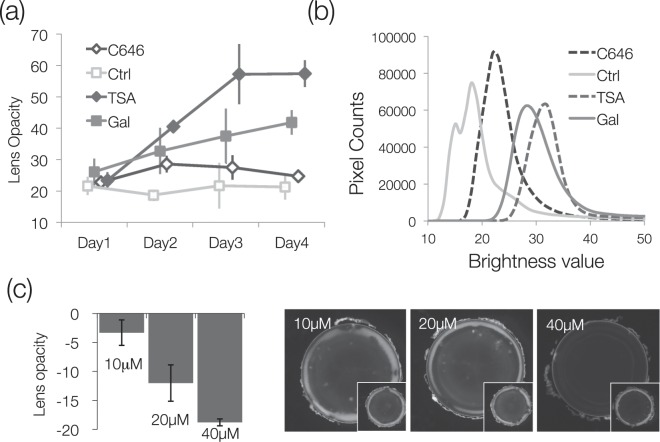
Effects of HAT/HDAC inhibition on lens opacity. (**a**) Changes in lens opacity over time. Opacity, which is a weighted average of lens brightness values, is sensitive to the effects of strong white turbidity in the cortex. C646 and TSA were added to galactose medium. All lenses on each date were removed from different animals (*n* = 3, only galactose is *n* = 9). Galactose-containing medium was used as the Ctrl (right lens). (**b**) A histogram of luminance values is shown to visualize the opacity of the entire lens. The same lens as Day 4 of Fig. 2a was used. (**c**) Concentration-dependent decreases in opacity with C646 treatment. The weighted average values of the left lens (inhibitor condition) to the right lens (galactose-only condition) of the same rat were subtracted. The right picture shows the effect of the C646 HAT inhibitor by concentration. The inset is the Ctrl (right) lens. Data are expressed as mean ± SD.

To quantitatively evaluate lens opacity, the average value of the brightness of the entire lens was calculated from the micrograph. We used C646 as a representative HAT inhibitor and TSA as an HDAC inhibitor, and measured turbidity daily on Days 1–4 of incubation. The Ctrl group, incubated in normal medium, did not change from Day 1 to Day 4, but turbidity increased in the galactose group in a time-dependent manner (Fig. 2a). There was no significant difference between the galactose, C646, and TSA groups on Day 1 (Fig. 2a). However, from Days 2–4, opacity increased in the galactose and TSA groups in a time-dependent manner, whereas in C646, opacity was similar to that of Day 1 (Fig. 2a). Next, to observe changes in lens luminance distribution, the lens histogram was calculated on Day 4 (Fig. 2b). This is strongly dependent on the strong turbidity of the cortex, and the brightness of the entire lens is illustrated in a single peak. This analysis also suggested that galactose and TSA increased lens opacity, while C646 decreased lens opacity (Fig. 2b). To investigate concentration-dependent effects of C646, we evaluated its effects at varying concentrations, identifying that turbidity gradually decreased from 10 μM to 40 μM (Fig. 2c). These results indicate that diabetic-like cataract formation is affected by histone acetylation, suggesting that recovery of histone acetylation levels attenuates cataract formation.

### Downstream factors controlled by HAT

**Figure 3:**
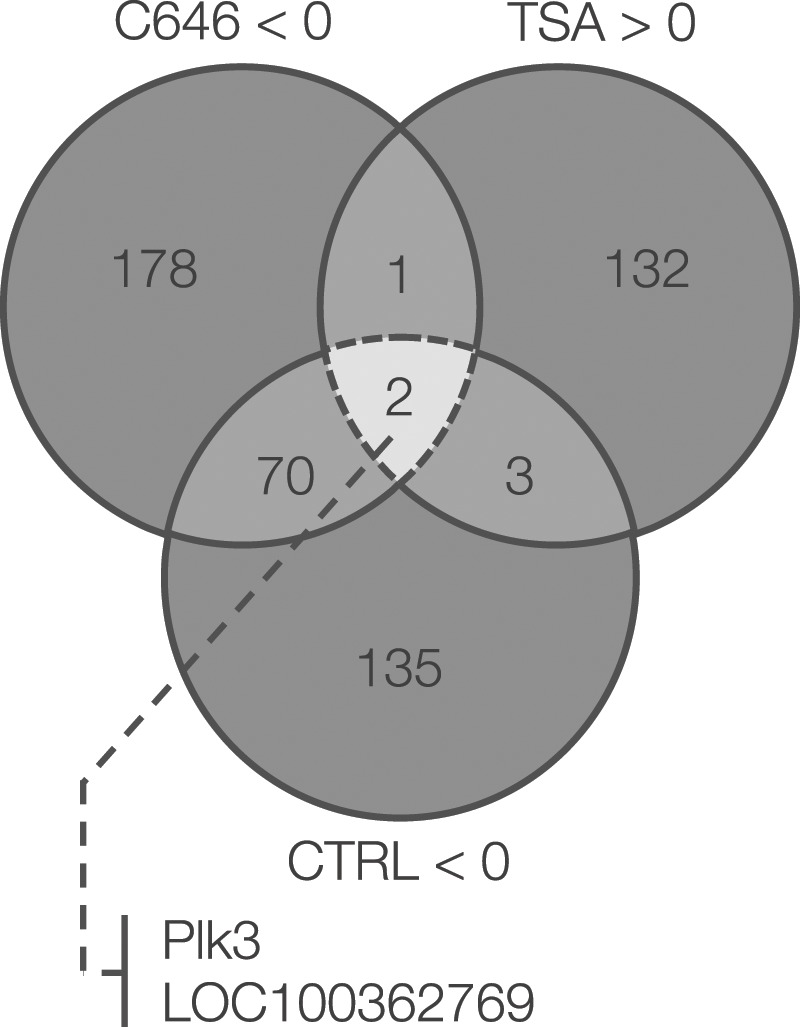
Identification of epigenetically regulated cataract-causing factors by microarray. Venn diagram of fluctuated genes in microarray analysis. C646 and Ctrl show *P < 0.05 and reduced gene clusters compared with galactose. TSA shows *P < 0.05 and an increased gene cluster compared with galactose.

Our findings suggested that HAT and HDAC might affect cataract formation, so we next sought to determine which downstream mediator(s) may be responsible for the observed effects. To comprehensively assess cataract-mediating factors oppositely regulated by HAT and HDAC, we performed a microarray. First, we created a Venn diagram with a group of genes significantly reduced in the Ctrl and HAT inhibitor (C646 40 μM) groups, and a group of genes significantly increased in the HDAC inhibitor group (TSA 0.8 μM) compared with galactose. There were 72 differentially regulated genes common between C646 and Ctrl, but there were five genes common between TSA and Ctrl, and three between TSA and C646. *Plk3* and *Loc100362769* were the only genes that were significantly decreased in C646 and Ctrl, and significantly increased in TSA (Fig. 3). Next, we performed annotation analysis using DAVID (https://david.ncifcrf.gov) to investigate whether the genes related to these two genes were affected by cataract formation. We uploaded the gene group (210) increased in the galactose group relative to the Ctrl group, and extracted the top three clusters with high enrichment scores (Table 2). Apoptosis and FoxO terms in the top 1 and 3 clusters of this list included *Plk3*.

**Table 2:** Annotation Cluster list.

Category	Term	Count	PValue	Fold Enrichment
**Annotation Cluster 1**	**Enrichment Score: 2.28**			
KEGG_PATHWAY	rno04068:FoxO signaling pathway	5	1.15E-03	10.22
KEGG_PATHWAY	rno04115:p53 signaling pathway	4	1.75E-03	15.88
KEGG_PATHWAY	rno04110:Cell cycle	3	7.12E-02	6.56
**Annotation Cluster 2**	**Enrichment Score: 2.09**			
GOTERM_BP_DIRECT	GO:0010628~positive regulation of gene expression	7	1.65E-03	5.42
GOTERM_BP_DIRECT	GO:0042493~response to drug	7	9.17E-03	3.81
GOTERM_BP_DIRECT	GO:0010629~negative regulation of gene expression	4	3.60E-02	5.42
**Annotation Cluster 3**	**Enrichment Score: 1.83**			
GOTERM_BP_DIRECT	GO:0043065~positive regulation of apoptotic process	6	5.93E-03	5.10
UP_KEYWORDS	Apoptosis	5	1.51E-02	5.20
GOTERM_BP_DIRECT	GO:0006915~apoptotic process	5	3.65E-02	3.93

Gene ontology analysis of 210 genes whose expression was significantly increased compared with the control. The top three enrichment scores are shown.

**Figure 4:**
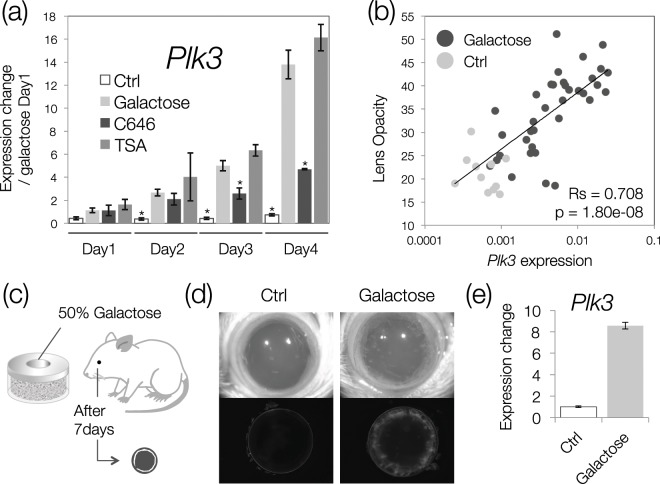
*Plk3* expression correlates with cataract severity. (**a**) *Plk3* mRNA expression over time was measured using real-time qRT-PCR (*n* = 3/group, galactose only *n* = 9). C646 and TSA were added to galactose medium. Data are expressed as mean ± SEM. *P < 0.05 versus galactose only. (**b**) The horizontal axis plots the logarithmic value of *Plk3* expression level, and the vertical axis plots the degree of lens opacity. Each point used galactose and control from Days 1–4. *Rs* and *P* value is cultured Spearman’s rank correlation. mRNA levels were normalized to *Gapdh* expression. (**c**) Diagram of the *in vivo* experiment. (**d**) Photomicrograph of the eye above, and of the lens removed below. (**e**) mRNA expression *in vivo* was measured using real-time qRT-PCR (n = 4).

**Figure 5:**
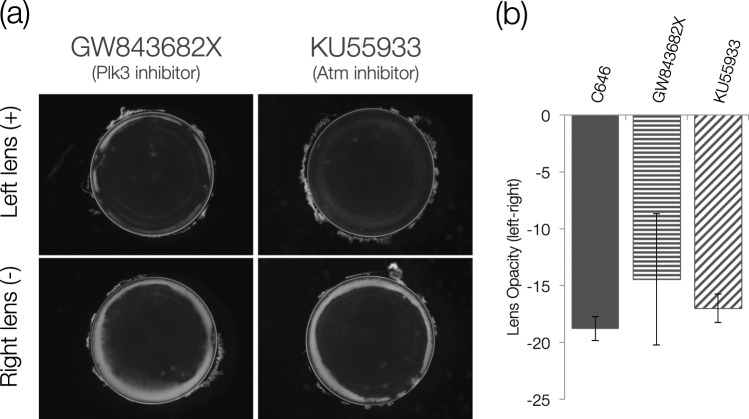
Plk3 and Atm inhibition alleviate galactose-induced cataract formation. (**a**) Representative photomicrographs of Day 4 lenses after inhibitor addition (Plk3 inhibitor GW843682X 1 μM, Atm inhibitor KU55933 10 μM). GW843682X or KU55933 was added to galactose medium. (**b**) Comparison of lens turbidity between inhibitors. The values of the left lens (inhibitor condition) to the right lens (galactose-only condition) from the same rat were subtracted. Data are expressed as mean ± SD.

To confirm the microarray results, *Plk3* expression was quantitatively measured by real-time qRT-PCR (Fig. 4a). We measured *Plk3* expression in all four groups from Days 1–4, identifying that *Plk3* expression increased in the galactose group relative to the Ctrl in a time-dependent manner (Fig. 4a). In the C646 group, *Plk3* expression was similar to that of the galactose group on Day 1, but gradually decreased after Day 2 in a time-dependent manner (Fig. 4a). *Plk3* expression was higher in the TSA group than in the galactose group at all time points, but this difference was not statistically significant (Fig. 4a). Furthermore, we compared the turbidity of Ctrl and galactose samples with *Plk3* expression, revealing a monotonically increasing relationship, suggesting that lens turbidity and *Plk3* expression were positively correlated (Fig. 4b). To demonstrate that these phenomena are not *ex vivo* specific, we performed *in vivo* experiments (Fig. 4c). As a result, cataracts induced by galactose-containing diets closely resembled those *ex vivo* and had increased *Plk3* expression (Fig. 4d,e). Taken together, these findings demonstrate that Plk3 is regulated by HAT and HDAC, and is associated with lens turbidity. To determine the causative role of Plk3 in cataract formation, we assessed the effect of Plk3 inhibitor in galactose-induced cataract formation. Cataract formation was decreased by treatment with the Plk3 inhibitor GW843682X (Fig. 5a, left). Plk3 is activated by the DNA damage response factor Atm^32^. Addition of KU55933, an Atm inhibitor, also decreased cataract formation (Fig. 5a, right). Both inhibitors had a similar efficacy to that of C646 (Fig. 5b).

### Downstream factors controlled by Plk3 and Atm inhibitors

**Figure 6:**
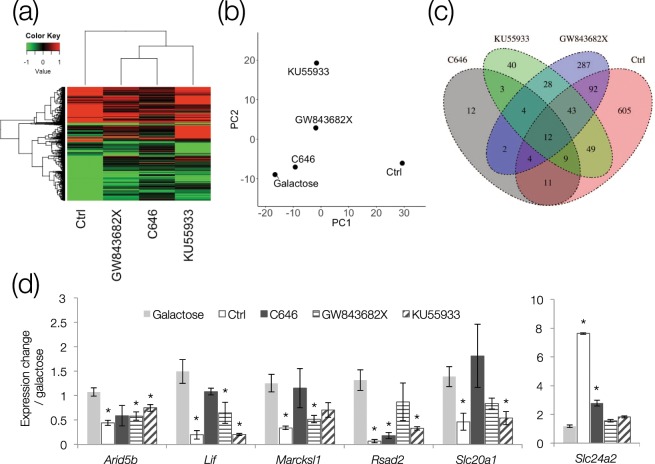
Correlation of HAT, Plk3, and Atm inhibitors. C646, GW843682X, or KU55933 was added to galactose medium. (**a**) Heat map of genes (1201) with more than a 2-fold variation in any one or more treatment group. (**b**) Principal component analysis map of 1201 genes. Proximal points in the plot have high expression profile homology. (**c**) Venn diagram of 1201 genes. In 12 genes that fluctuated more than 2-fold under all conditions, the increasing and decreasing tendencies agreed. (**d**) Real-time qRT-PCR of six genes (five reduced genes and one increased gene) that showed significant expression changes in any sample compared with galactose. mRNA levels measured were normalized to *Gapdh* expression levels. Data are expressed as mean ± SE. *P < 0.05 versus galactose.

To investigate the relationships between Hat, Plk3, and Atm inhibitors, we performed microarray analysis. A total of 1201 genes with two-fold or more changes in expression compared to the control (galactose) following treatment with each inhibitor were extracted. The increasing and decreasing patterns of expression in many genes were similar between the three inhibitor treatments (Fig. 6a). Cluster and PCA analyses identified high similarity between the genes inhibited by C646 and GW843682X, but the Ctrl was located in the furthest cluster (Fig. 6a,b). Because there was no significant trend in the overall expression pattern, we sought to determine whether the three inhibitors commonly affected the expression of a subset of genes. When the heat map in Fig. 6a was replaced with a Venn diagram, 12 genes showed variations in expression between the Ctrl group and each of the three inhibitor groups (Fig. 6c). The results of gene cluster analysis were confirmed by real-time qRT-PCR, and six genes with significant differences in expression irrespective of the inhibitor used were extracted (Fig. 6d). *Arid5b*, *Lif*, and *Rsad2* had significant differences in expression, suggesting that these genes are common downstream factors of HAT, Atm, and Plk3.

## Discussion

In diabetic cataracts, accumulation of sorbitol increases osmotic stress and apoptosis, disrupting lens homeostasis and ultimately leading to cataract formation^4,5^. However, the detailed molecular mechanisms of diabetic cataract remain incompletely understood. In the present study, we report that HAT inhibition alleviated galactose-induced diabetic-like cataract formation, and demonstrated that histone acetylation correlates with cataract formation. Furthermore, microarray analysis identified that *Plk3* was controlled by HAT, and subsequent functional analyses revealed that Plk3 contributed to galactose-induced cataract formation. These findings represent a novel mechanism of epigenetically regulated diabetic cataract formation.

Plk3 is a serine/threonine kinase, and plays a role in cell cycle progression and tumorigenesis^33^. Plk3 is activated by Atm and causes apoptosis via p53 activation^32,33^. Although no prior studies have reported a direct relationship between Plk3 and cataract, exhaustive gene expression analyses of the murine LEGSKO and Mip mutant cataract models revealed that *Plk3* expression was increased in both models compared with WT^34,35^. Also, some reports indirectly support our hypothesis that Plk3 regulated cataract formation. For example, the cataract-inducing factors H_2_O_2_ and UV both activate Plk3^36,37^. UV-induced cataract is attenuated by caffeine, which also inhibits Plk3 via Atm^32,38,39^. In addition, osmotic stress is also a risk factor for cataract formation, and activates Plk3. Further, Wang *et al*. revealed that in human corneal epithelial cells osmotically stressed by excess sorbitol, DNA damage occurs and Plk3 is activated^40^. In diabetic cataracts, osmotic pressure is increased by polyol produced from sugar, and our results demonstrate that Plk3 is up-regulated in the context of galactose-induced diabetic cataract^41^ (Fig. 4a).

Several reports have previously identified that HDAC is activated in age-related and Tgf-β induced cataracts, and that cataract formation is alleviated by HDAC inhibition, unlike in the present study^14,18,19^. However, there are no prior reports of HDAC activity is involved in diabetic or galactose-induced cataract formation. In particular, Tgf-β-induced cataract is caused by multilayered LECs, and the mechanism of cataract formation is fundamentally different^42^. Also, in diabetic retinopathy, histone acetylation is increased, and HDAC activation alleviates the pathology of retinopathy^43^. Therefore, it is possible that ocular histone acetylation increases in diabetes and in ocular diabetic complications. *Plk3* may also be controlled by epigenetics in this context.

The tumor suppressor p53 is a transcription factor that binds p300, inducing apoptosis. p53 overexpression not only up-regulates Plk3, but also activates histone acetylation of the *Plk3* promoter^44^. However, these findings were obtained in cancer cells, so they may not be relevant to the crystalline lens. Therefore, it will be necessary to confirm this effect of p53 in crystalline lens, and determine whether p53 inhibition has a similar functional effect to that of Plk3 inhibition.

We also sought to determine which factors operate downstream of Plk3, and identified several factors including Arid5b, Lif, and Rsad2 (Fig. 6d). Interestingly, these factors are involved in lipid metabolism, which is surprising because there has been no report so far about a link between lipid metabolism and cataract. Arid5b is a regulator of vascular smooth muscle cell differentiation and proliferation^45^. It complexes with the demethylase Phf2 to function as an epigenetic determinant of signal sensing by removing repressive histone methylation marks from to its target promoters^46^. Phf2-overexpressing mice develop steatosis, and *Arid5b* knockout mice are resistant to high fat diet-induced weight gain and obesity^47,48^. Lif induces terminal differentiation of myeloid leukemia cells, and inhibits their growth^49^. It also reduces the activity of lipoprotein lipase, an enzyme responsible for triglyceride degradation, through transcriptional regulation^50^. Administration of Lif dose-dependently increases rat serum triglyceride levels^51^. Rsad2 is found in a wide range of organisms, and inhibits viral replication^52^. Genotype-phenotype correlations in obese mice using ATR-FTIR spectroscopy has shown that Rsad2 is associated with obesity-related diseases^53^. Additionally, *Rsad2* knockdown mice show increased inflammation of adipose tissue caused by decreased antiviral activity, but are immune to the deleterious effects of high fat diet feeding^54^. Thus, inhibition of Arid5b, Lif, and Rsad2 may be effective in reducing diabetes-related disorders, such as the impaired homeostasis of lens epithelial cells. Determining the roles of these three factors in lens opacity will require further investigation.

The present study demonstrated for the first time that Plk3 upregulation through HAT may be involved in the onset of diabetic cataract. This suggests that inhibitors of HAT, Plk3, and Atm are potential therapeutic modalities for diabetic cataract. The future work, we will need to find for regulator of HAT and direct regulator of Plk3.

## Materials and Methods

### *In vivo* and *Ex vivo* assays

Six-week-old male Sprague Dawley rats were purchased from Sankyo Laboratory Service. Rats were euthanized with CO_2_ asphyxiation, and eyeballs were removed. For *Ex vivo* experiments, whole lenses were maintained in 2 ml serum-free M199 medium containing 0.1% BSA^55^. C646 (Wako), TSA (Wako), GW843682X (AdooQ Bioscience), or KU55933 (Chemscene) were added to the culture medium at the final concentrations of 40, 0.8, 1, and 10 mM, respectively. All lenses were maintained at 37 °C in a humidified incubator with 95% room air and 5% CO_2_. Culture medium (Sigma) was renewed every second day throughout the culture period. Lenses were cultured for up to 4 days and photographed using a microscope. For *in vivo* experiments, rats were fed the following diets for 1 week: normal MF powder (Ctrl), and MF powder plus 50% galactose (Galactose). The rats were then anesthetized the rats and their eyes were photographed via a microscope. The experiments were approved by Animal Research Committee, University of Fukui (Application number: 28091), and all experiments were performed in accordance with relevant regulations for Animal Research at University of Fukui.

### Microscopic observation

Images were captured in the dark using a SZX12 stereomicroscope combined with a DP58 camera (Olympus). At this time, the lens was placed in a 35 mm dish containing 7 mL PBS. The degree of opacity was measured as brightness (~0–255) using ImageJ, and a weighted average was calculated.

### RNA extraction, cDNA preparation, and real-time qRT-PCR

After culture, the entire lens was homogenized in TRIzol reagent (Thermo Fisher Scientific) and RNA was extracted according to the manufacturer’s instructions. cDNA was synthesized with a reverse transcription kit (Applied Biosystems). For quantitative analysis of mRNA expression, SYBR Green master mix (Applied Biosystems) was used to amplify the target genes and *Gapdh*.

### Tissue sectioning and staining

After incubation, the lenses were immersed in a formalin-glutaraldehyde (FG) solution at 4 °C for 3 days as described previously^56^. FG solution was exchanged every other day. The lenses were then left in 10% formalin solution for at least 1 day at room temperature.

### Microarray data analysis

All the samples used for microarray were lenses collected at Day 4. We used a GeneChip Rat Gene 2.0 ST Array (Thermo Fisher Scientific) for microarray analysis. We excluded unnamed genes, and genes with a max signal value below 5. Each condition’s signals were normalized by the galactose-treated sample’s signal values. Genes with significant expression changes (p < 0.05) in galactose-treated lenses were used for GO term analysis. GO term analysis was performed in the DAVID database (https://david.ncifcrf.gov). Heat mapping was conducted for genes that varied more than twice as much as galactose-treated samples in any one of the respective conditions.

### Statistical analysis

Dunnett’s t-test was used to determine the difference in averages between Ctrl and experimental groups. P < 0.05 was considered statistically significant. The coefficient correlation was calculated by Spearman’s method. Statistical analyses were performed using R.

## Supplementary information

Dataset1,2
