# Supplementary material for: Histone acetyltransferase and Polo-like kinase 3 inhibitors prevent rat galactose-induced cataract
Source: Sci Rep. 2019 Dec 27;9:20085. doi: 10.1038/s41598-019-56414-x (PMC6934598; doi:10.1038/s41598-019-56414-x)
Supplement: Supplementary file 1 — Dataset1,2 [file 41598_2019_56414_MOESM1_ESM.pdf]

Histone acetyltransferase and Polo-like kinase 3 inhibitors prevent rat galactose-induced cataract

Fumito Kanada[1], Yoshihiro Takamura[2], Seiji Miyake[2], Kazuma Kamata[1], Mayumi Inami[1], Masaru Inatani[2] & Masaya Oki[1,3]

[1]Department of Applied Chemistry and Biotechnology, Graduate School of Engineering, University of Fukui, Fukui 910-8507, Japan; [2]Department of Ophthalmology, Faculty of Medical Sciences, University of Fukui, Fukui 910-1193, Japan; [3] Life Science Innovation Center, University of Fukui, Fukui 910-8507, Japan.

Correspondence and requests for materials should be addressed to M.O.(email: ma4sa6ya@u-fukui.ac.jp)

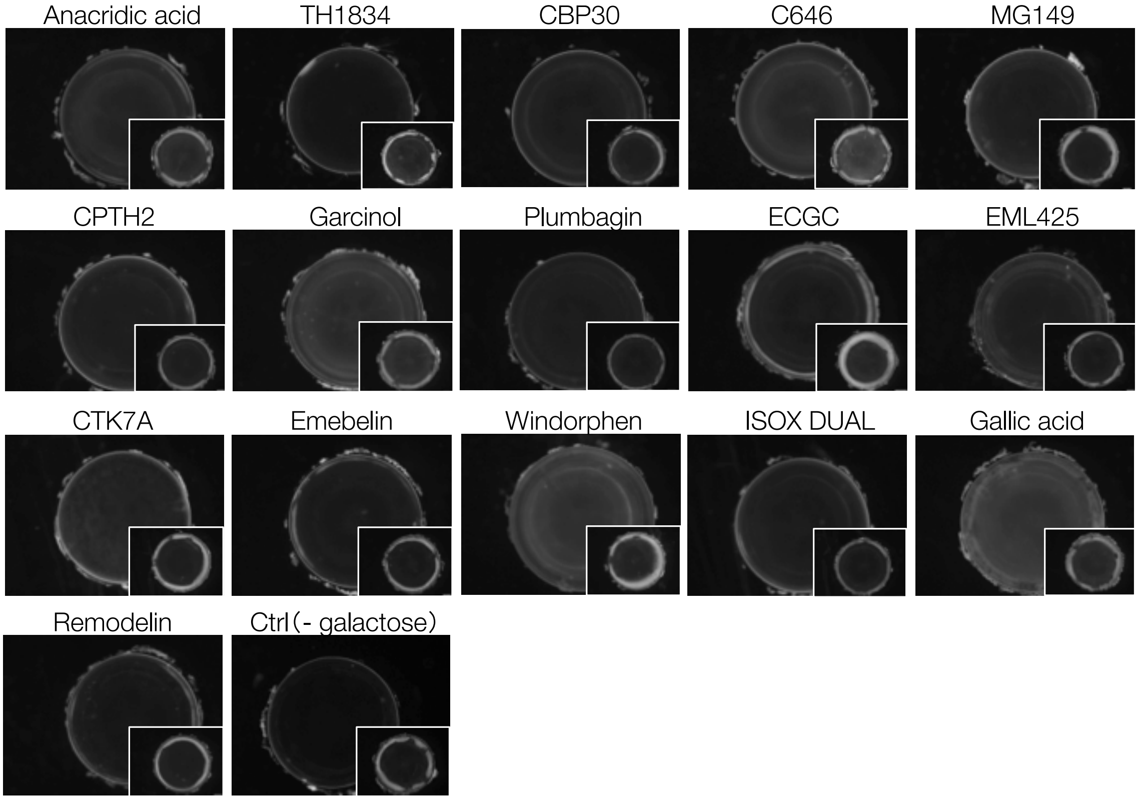

Supplementary Figure 1. HAT inhibitor with a cataract preventive effect . A photomicrograph of a day 4 lens after addition of HAT inhibitors. A small picture on the bottom right shows the right lens of the control.

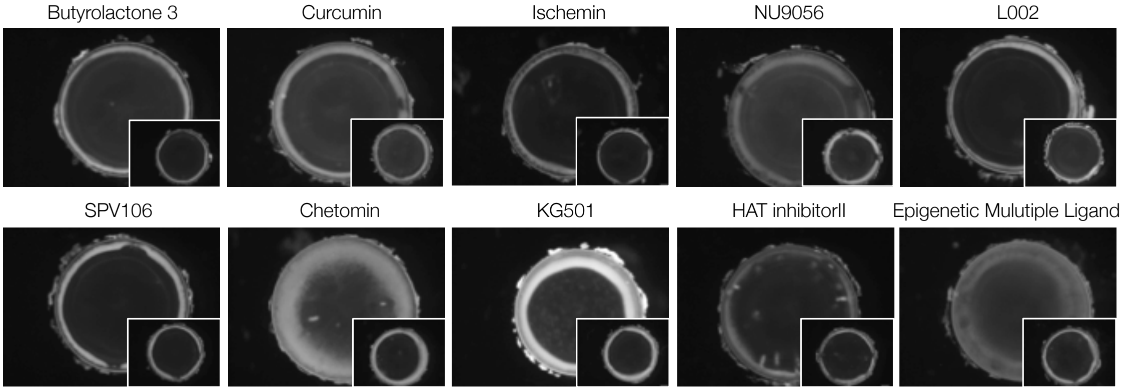

Supplementary Figure 2. HAT inhibitor with no preventive effect. A photomicrograph of a day 4 lens after addition of HAT inhibitors. A small picture on the bottom right shows the right lens of the control.
